# Temperature-dependent yield stress and wall slip behaviour of thermoresponsive Pluronic F127 hydrogels†

**DOI:** 10.1039/d4ra04825c

**Published:** 2024-07-29

**Authors:** Surya Narayana Sangitra, Ravi Kumar Pujala

**Affiliations:** a Soft and Active Matter Group, Department of Physics and Center for Atomic, Molecular and Optical Sciences & Technologies (CAMOST), Indian Institute of Science Education and Research (IISER) Tirupati Yerpedu Tirupati 517619 Andhra Pradesh India pujalaravikumar@iisertirupati.ac.in

## Abstract

This study explores the temperature-dependent dynamic yield stress of a triblock thermoresponsive polymer, Pluronic F127, with chemical structure (PEO)_100_(PPO)_65_(PEO)_100_, during the sol–gel transition. The yield stress can be defined as static, dynamic, or elastic, depending on the experimental protocol. We examine the dynamic yield stress estimation for this study, which usually entails utilizing non-Newtonian models like the Herschel–Bulkley (HB) or Bingham models to extrapolate the flow curve (shear rate against shear stress). Initially, we determine the yield stress using the HB model. However, apparent wall slip makes it difficult to calculate yield stress using conventional methods, which could lead to underestimates. To validate the existence of apparent wall slip in our trials, we carry out meticulous experiments in a range of rheometric geometries. To determine the true yield stress corrected for slip, we first use the traditional Mooney method, which requires labor-intensive steps and large sample sizes over various gaps in the parallel plate (PP) design. To overcome these drawbacks, we use a different strategy. We modify the Windhab model equation by adding slip boundary conditions to the HB equation, which allowed us to calculate the slip yield stress in addition to the true yield stress. In contrast to other typical thermoresponsive polymers like poly(*N*-isopropyl acrylamide) (PNIPAM), our findings demonstrate that PF127's yield stress obeys the Boltzmann equation and increases with temperature.

## Abstract 2

A new method to calculate slip yield stress along with true yield stress is proposed in thermoresponsive hydrogels.

## Graphical abstract

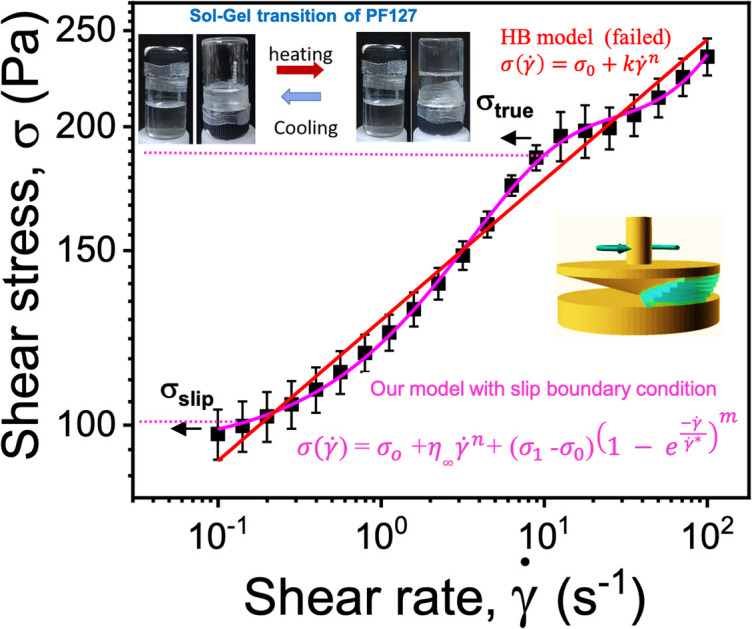

## Introduction

Hydrogels have become a highly adaptable material with a wide range of uses in many different domains, such as biomedical engineering, tissue engineering, and drug delivery.^1^ Because of their exceptional capacity to experience sol–gel transitions in response to temperature fluctuations, thermoresponsive hydrogels have been used in biomedical applications for over two to three decades.^2,3^ Poloxamers are the second most commonly used thermoresponsive polymer after poly(*N*-isopropyl acrylamide) (PNIPAM). Poloxamers are typically non-ionic tri-block copolymers in the form of PEO–PPO–PEO based on a hydrophilic block of poly(ethylene oxide) (PEO) and hydrophobic block of poly(propylene oxide) (PPO).^3^ Commercially, poloxamers are also known as Pluronics. Based on molecular weight and physical state of Pluronic, it is available in different forms such as L31, P104, P85, F127, *etc.*, where the first letters L, P, and F represent the physical form commercially available at room temperature as liquid, paste and flaked solid, respectively and numbers represents the PPO molecular mass and PEO content.^4^ F127 has been used for various biomedical applications because its sol-to-gel transition temperature is close to body temperature, and its rheological properties play an essential role in different biomedical and drug delivery applications.^5^

Hydrogels are intricate materials that exhibit neither simple liquid nor perfectly elastic solid behavior. They exhibit viscoelastic behavior with both elastic and viscous components and have mechanical behavior that differs from that of solids and liquids. As the lowest stress necessary for the material to flow, the yield stress is a critical parameter in describing the mechanical behavior of hydrogels. Since it controls hydrogel-based systems' injectability, stability, and flow characteristics, yield stress is crucial in these applications.^6–8^

Different methods have been proposed to determine the yield stress. The yield stress can be defined as static, dynamic, or elastic, depending on the experimental protocol.^9,10^ The static and elastic yield stress can be found directly from creep and oscillatory shear experiments, respectively, while dynamic yield stress can be calculated by extrapolating the flow curve (shear rate *versus* shear stress) by fitting with non-Newtonian models such as Bingham or HB model, *etc.* Minimum stress at which, if one can wait sufficient time, the sample reaches a final steady state with constant viscosity is defined as static yield stress, and above static yield stress, the sample shows viscosity bifurcation. The creep rheology test can find this phenomenon. Similarly, the elastic yield stress can be calculated from oscillatory amplitude sweep measurements by measuring the deviation from linearity, where plastic deformation begins and the sample no longer fully recovers. The dynamic yield stress calculated from flow curves measures the minimum stress needed to maintain flow.^10^

Here, we aim to investigate the temperature-dependent dynamic yield stress of PF127 during the sol–gel transition. Surprisingly, the traditional method of calculating dynamic yield stress by fitting the HB model for the whole range of data could not help us with different temperatures. Following a comprehensive review of the literature, we found that apparent wall slip can underestimate true yield stress values and is one of the difficulties in computing the dynamic yield stress.^11^ Therefore, accurately determining the yield stress of hydrogels is crucial for optimizing their performance and understanding their rheological behavior.^12^ The following describes the origin and history of the apparent wall slip.

The rheological characterization of complex materials, such as colloidal gels or soft microgels and others, changes when bounded on smooth surfaces compared to rough or serrated surfaces due to wall effects.^13^ The effects of walls in complex fluids are often interpreted as apparent wall slip. Wall slip can be well understood from a typical example when a solid block is kept between two parallel plates with smooth polished surfaces, one of which is in motion, and it is due to inadequate friction present on the smooth surface.^14^ Similar situations can also arise in various complex fluids when they exhibit solid-like behavior under external conditions like time, temperature, shear rate, *etc.* For example, some complex materials behave like liquids at a low shear rate and exhibit solid-like behavior at a higher shear rate.^14^ In some cases, solid-like behavior is observed after a specific time, keeping the temperature and other parameters constant.^10^

For most complex materials, apparent slips in dispersion flow are described by forming a thin depletion layer (nm to μm) close to the bounded wall.^11–13,15–21^ The layer may consist of pure solvent or deficient particle concentrations compared to the bulk dispersion. The literature shows a high-velocity gradient near the wall due to an apparent slip. The velocity profile in between the rheometric plate gap can be directly measured by several advanced techniques combined with conventional rheometers such as particle image velocimetry (PIV),^22^ nuclear magnetic resonance (NMR) velocimetry,^23^ ultrasonic speckle velocimetry (USV)^24^ and dynamic light scattering (DLS), *etc.*^25^ One can also indirectly observe the wall slip from flow curves (shear rate *versus* shear stress).^13^ Over the years, a large number of studies have investigated wall slip in complex fluids in the literature. While it is difficult to include them all in Table 1, we have documented a few of them. Nevertheless, several important review papers give a historical perspective and describe the advancements made in understanding and dealing with wall slip so that the yield stress may be accurately calculated.^10,15–18,20,21,25–29,37–40^

**Table 1:** List of articles published on wall slip behaviour and their significance in different systems

Publication	Summary	Characteristic quantities	Advantages	Disadvantages
Mooney (1931)^30^	Indirect quantification of wall slip for rotational and capillary viscometry	Slip velocity profiles, shear rate dependencies	Established foundational concepts	Requires three different sets of measurements, time-consuming and requires precise instrumentation
Yoshimura *et al.* (1988)^31^	Wall-slip corrections for Couette cylinders and parallel disk viscometers	Correction factors, experimental validations	Improved measurement accuracy	Specific to certain geometries
Yilmazer *et al.* (1989)^32^	Improved wall-slip corrections using four sets of gaps between parallel plates	Gap size dependencies, empirical data	Enhanced reliability	Requires multiple gap sizes
Hatzikiriakos *et al.* (1992)^33–35^	Study of wall-slip of molten high-density polyethylene (HDPE) using capillary and sliding plate rheometer	Slip coefficient values, temperature effects	Comprehensive polymer data	Complex experimental setup
Aral *et al.* (1994)^36^	Effect of surface roughness and temperature on the transient behavior of wall slip in concentrated suspensions	Roughness parameters, transient slip data	Detailed surface interactions	Limited to specific suspensions at high concentration
Rosenbaum *et al.* (1997)^37^	Effect of viscous heating on high shear rheological measurements using various polymers	Temperature profiles, shear heating effects	Applicable to high-shear processes	Requires thermal control
Bertola *et al.* (2003)^23^	Generalized method with seven different plate gaps, validated with magnetic resonance imaging (MRI) in pasty materials	MRI imaging data, multi-gap analysis	High-resolution slip data	Requires multiple measurements and time-consuming
Meeker *et al.* (2008)^38,39^	Proposal of a mechanism using micro-elastohydrodynamic lubrication theory for slip and flow in soft particle pastes	Lubrication theory, stress–strain curves	Theoretical and fundamental insights for soft particle system	Valid for soft particle system and does not include role of short-range force in the theory
Seth *et al.* (2008)^40^	This work investigate the role of short range forces on wall slip behaviour of microgel paste	Role of short-range forces on wall slip behaviour	This study expands on the elastohydrodynamic slip model developed by Meeker and colleagues by including attractive or repulsive forces between the slipping paste particles and the wall	Valid for soft particle systems and need validation for hard colloidal particle system
Manneville *et al.* (2008)^24^	Discussion on wall slip and flow instability of Pluronic P84 in brine solutions	Instability maps, slip transition points	Detailed instability analysis	Specific to Pluronic P84
Wang *et al.* (2010)^41^	Study on homogeneous shear, wall slip, and shear banding of entangled polymeric liquids in simple-shear rheometry	Shear banding profiles, stress distributions	Detailed shear banding analysis	Limited to entangled polymeric liquids
Ballesta *et al.* (2013)^42^	Investigation of slip behavior in colloidal gels formed under polymer-induced depletion attraction, including time-dependent rheology	Time-dependent slip data, gel behavior	Time-resolved analysis and valid for hard sphere colloidal system	Limited to specific colloidal gels
Divoux *et al.* (2015)^43^	Study on wall slip across the jamming transition of soft thermoresponsive particles	Jamming transition data, stress–strain analysis	Comprehensive particle behavior	Requires controlled conditions
Chatzigiannakis *et al.* (2017)^44^	Study on wall slip of polyisobutylenes and its relationship with molecular characteristics	Molecular interaction data, slip velocities	Detailed molecular insights	Specific to polyisobutylenes
Chin *et al.* (2019)^45^	Development of a rheological wall slip velocity prediction model based on artificial neural network	Artificial neural network (ANN) model predictions, validation data	Predictive modeling and time efficiency	Requires ANN expertise and experimental verification for different system
Georgiou (2021)^46^	Analysis of simple shear flow of Herschel–Bulkley fluids with wall slip above a threshold stress	Slip threshold data, HB fluid behavior	Theoretical framework	Needs experimental support (only discussed about parallel plate geometry)
Moud *et al.* (2021)^47^	Characterization of wall slip in colloidal suspensions of kaolinites	Slip characterization data, suspension behavior	Detailed suspension analysis and new slip model	Limited to kaolinites clays
Moud *et al.* (2022)^48^	Study on apparent slip in colloidal suspensions	Apparent slip profiles, rheological data	Comprehensive slip analysis	Temperature dependency not discussed
Our work	Characterize the temperature-dependent yield stress and wall slip behavior of Pluronic F127	Yield stress values, fitting the steady state flow curve using a non-Newtonian model	Simple and easy method to calculate the true dynamic yield stress for any soft material	—

However, we were inspired by recent work by Moud *et al.*, which evaluated apparent slip in the colloidal suspensions of kaolinite clay and proposed a generalized slip model that can be applied to many other colloidal systems.^47^ This paper has not discussed the temperature-dependent behavior of thermoresponsive materials. However, Aral and Kalyon studied the time and temperature-dependent behavior of poly(butadiene–acrylonitrile–acrylic) acid terpolymers and found that the temperature-dependent yield stress decreases with increasing temperatures.^36^ Jalaal *et al.* performed a systematic study to understand the rheological phase behavior of PF127 by analyzing the flow curves at different temperatures and found that the yield stress increases as an increasing function of temperature.^49^ They used sandblasted parallel plates to avoid the wall-slip effect and calculated the yield stress by fitting with the Herschel–Bulkley (HB) model.^49^ But they calculated the yield stress for PF127 for different concentrations at 5 °C intervals, and from their experiments, it was shown that for thermo-responsive hydrogels like PF127, with short intervals such as 1 °C is crucial. Therefore, we investigate the temperature-dependent yield stress of PF127 gels with 1 °C during the sol–gel transition. Careful observation confirms the presence of apparent slip during the experiments.

Traditionally, there are three approaches to deal with the apparent slip while calculating the yield stress; first, the slip can be avoided by using surface modification or serrated plate or alternative geometries like vane-in-cup and helix to avoid the slip. Unfortunately, we could not avoid the slip from experiments due to experimental limitations. The second approach to calculate the true yield stress is by using Mooney's calculation. We have extensively calculated the true yield stress following Mooney's method. The third approach is to use a suitable model or theory that can include the slip and calculate the true yield stress. In this article, we modified the HB model by including the slip boundary condition and were able to calculate the true yield stress at different temperatures. Finally, we plot the dynamic yield stress as a function of temperature that was obtained using three different methods (the HB model, Mooney's plot, and our approach) and found that the yield stress of PF127 increases as a function of temperature and follows Boltzmann's equation for sigmoid curves. We also compare the yield stress of PF127 with other thermoresponsive materials such as poly(*N*-isopropyl acrylamide) (PNIPAM) and compare the results with PF127.

## Materials and methods

### Materials

We used Pluronic F127 ((PEO)_100_(PPO)_65_(PEO)_100_) with an average molecular weight (∼12 600 g mol^−1^) (Sigma-Aldrich) without further chemical modifications. Different concentrations of PF127 (1–35% (w/v)) are prepared using an ice bath following the cold technique in deionized (DI) water.^4^

### Methods

#### Rheology

Rheological measurements of PF127 samples were performed on an MCR 302 rheometer (Anton Paar) with stainless steel cone-plate geometry (CP) and parallel plate geometry (PP) with different diameters (CP-25, CP-60, PP-40, PP-50) in rotational and oscillatory modes. The oscillatory frequency sweep experiments were performed in the linear viscoelastic region (LVR) at constant shear stain amplitude *γ* = 0.1%, with angular frequency (*ω*) range from 0.1 to 100 (rad s^−1^) for different temperatures (20–26 °C) during the sol–gel transition to study the linear viscoelastic properties of PF127. The flow curve is plotted for rotational tests for different temperatures varying from 10 to 40 °C. The flow curves were measured by ramp-up in logarithmic scale in two input shear rates, one from 0.1 to 100 s^−1^ and another from 0.01 to 1000 s^−1^. The measuring point duration is kept in variable logarithmic scale with initial time, *t* = 100 s, and final *t* = 5 s. This approach aims to prolong the measuring points at lower shear rates while shortening them at higher rates, ensuring that transient effects of the sample are minimized or fully decayed by the end of each prolonged measuring point within the low-shear range. All experiments were carried out for PP40 with a plate gap size of 1 mm except otherwise specifically not mentioned. For calculating the true yield stress following Mooney's method, we also performed measurements at different gap sizes varying from (0.1, 0.25, 0.5, and 1 mm). To confirm the wall slip phenomena in our system, we also analyzed the steady-state flow curves at different geometries (CP-25, CP-60, PP-40, PP-50) at different temperatures.

## Results and discussions

### Linear viscoelasticity of PF127 at different temperatures

**Fig. 1:**
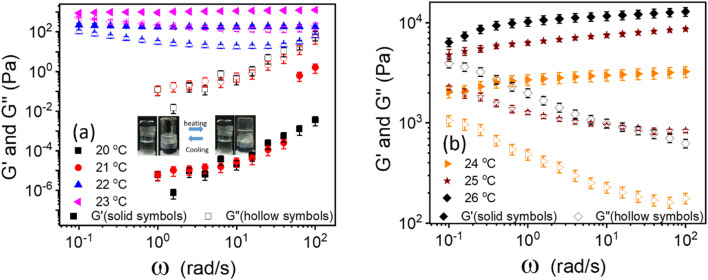
(a and b) Frequency-dependent moduli storage modulus (*G*′) and loss modulus (*G*′′) in the linear viscoelastic region (shear strain (*γ* = 0.1%)) for 20 wt% PF127 at varying temperatures from 20 to 26 °C.

The linear viscoelastic properties of 20% PF127 gels were investigated by frequency sweep experiments at a minimal shear amplitude strain, *i.e.*, at *γ* = 0.1%. The frequency sweep data for 20% PF127 at different temperatures is plotted in Fig. 1. For 20 and 21 °C, loss modulus (*G*′′) is dominant over storage modulus (*G*′) and for almost all frequency ranges. This represents the liquid-like (viscous) behavior of PF127. At low temperatures, the PF127 solution exhibits liquid-like behavior, and at 22 °C and above, the storage modulus (*G*′) is dominant over the loss modulus and indicates the viscoelastic fluids. At 22 °C, storage modulus (*G*′) shows a plateau at *G*′ = 200.2 ± 20.2 Pa, known as an elastic plateau in linear rheology. As the temperature increases, the elastic plateau values increase, and the sample behaves as viscoelastic solids, *i.e.*, gels. We found that these samples exhibit yield stress for the steady shear rheology (flow curves), and we calculated the dynamic yield stress in the following section.

### Determination of dynamic yield stress from steady-state flow curves

**Fig. 2:**
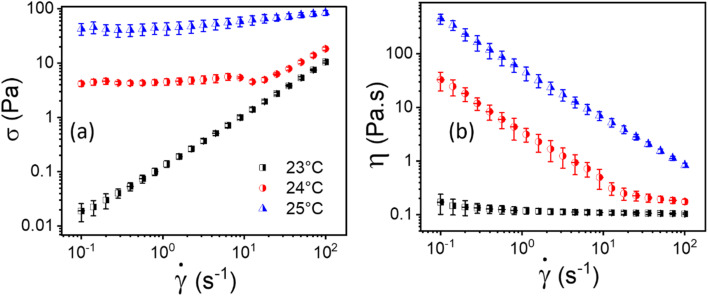
Flow curves, *i.e.*, (a and b) represents shear stress (*σ*) and viscosity (*η*) as a function of shear rate (*γ̇*), respectively, for 20% PF127 at varying temperatures of 23 °C, 24 °C and 25 °C.

The flow curves, *i.e.*, shear stress (*σ*) and viscosity (*η*) as a function of shear rate (*γ̇*) of 20% PF127 at temperatures 23 °C, 24 °C, and 25 °C are shown in Fig. 2. As shown in Fig. 2(a), For 23 °C, shear stress (*σ*) increases linearly as a function of shear rate (*γ̇*), which is typical Newtonian behavior, and this is also reflected in Fig. 2(b); the viscosity is independent of the shear rate (*γ̇*). The samples exhibit yield stress for 24 °C and 25 °C, which can be calculated using a non-Newtonian model. We defined this temperature as the sol–gel transition temperature using the same convention as Jalaal *et al.*^49^ (here, we have used a 20% PF127 sample, and other concentrations of PF127 are shown in ESI Fig. S1 and S2†).

**Fig. 3:**
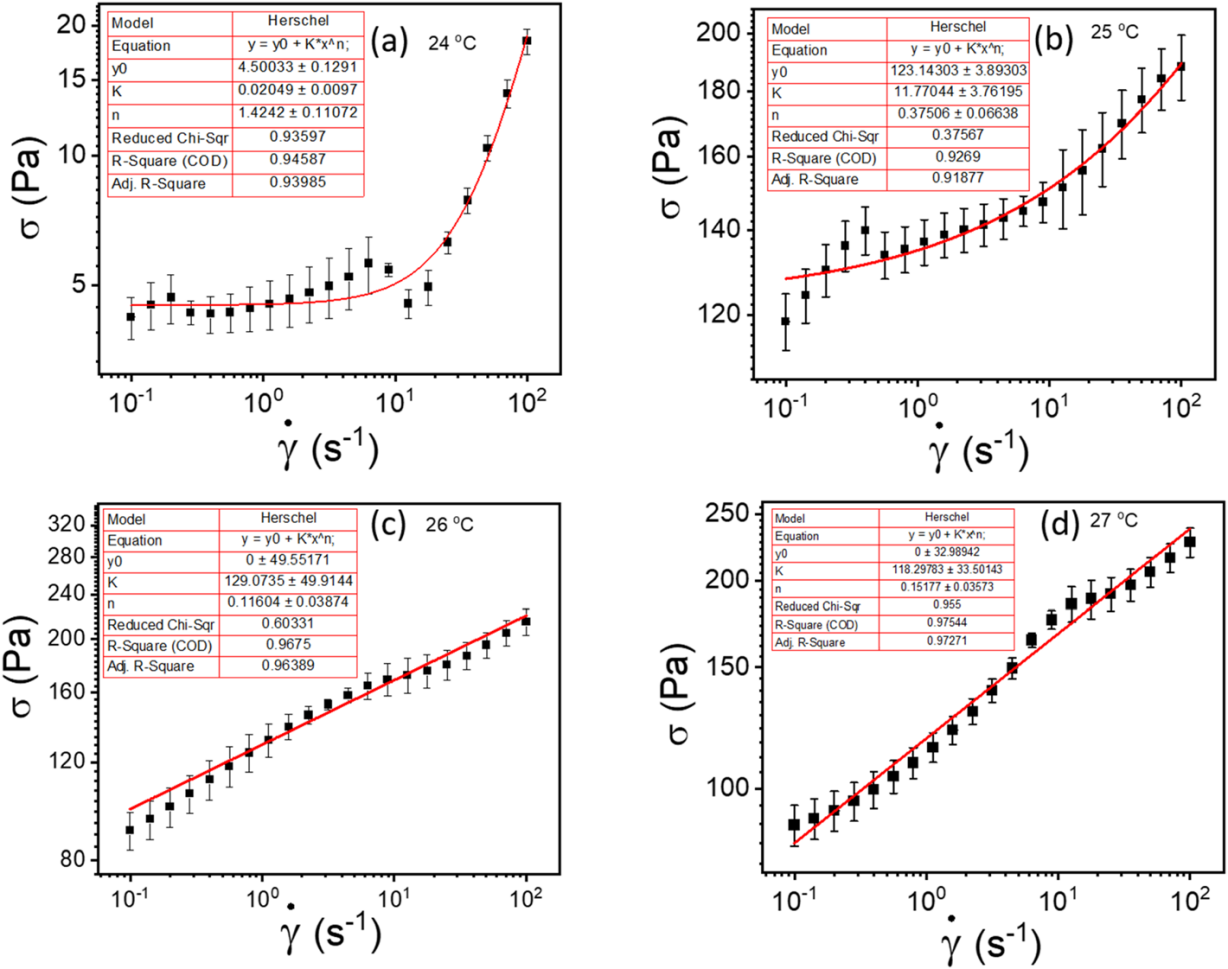
(a–d) represents HB model fitting of flow curves for 20% PF127 at temperatures varying from 23 to 27 °C, respectively.

For this work to calculate the yield stress, we have used the well-known Herschel–Bulkley (HB) model. The HB model with the constituent equation is given as follows,1
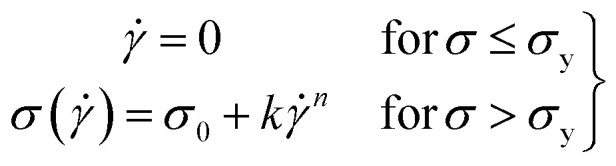
where *σ* is the shear stress, *γ̇* is the shear rate, *σ*_y_ is the yield stress, *k* is the consistency index, and *n* is the flow index. The HB model is fitted to the flow curves at different temperatures, and from Fig. 3, we observed that the HB model explains the data only for 24 °C and 25 °C (Fig. 3(a) and (b)). For 26 °C and 27 °C, it fails to explain the data (the yield stress (*y*_0_) value in the fitting parameter table shown in the figure index shows 0 ± 49.5 and 0 ± 32.9, for 26 °C and 27 °C, respectively, which has no meaning), and, we observe a slope change in the mid shear range for both data. Therefore, to understand these results, we took help from literature, and in this context, we found one exciting work by Georgios C. Georgiou.^46^ In his paper, they generated the flow curves numerically for parallel plate configuration by considering various aspects of wall slip.

They discussed that the following slip law must be employed to calculate true yield stress when a wall slip occurs during the measurements.2
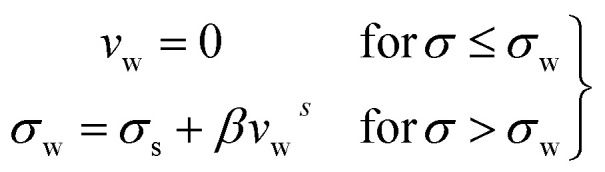
where, *v*_w_ is the relative velocity of the fluids with respect to the wall, *σ*_w_ is the wall shear stress, *σ*_s_ is the slip yield stress, *β* is the slip coefficient, and *s* is the slip exponent. Fig. S3† summarizes their works. Fig. S3(a and c)† represents flow curves shear stress (*σ*) *vs.* shear rate (*γ̇*) generated numerically for HB fluids in case of the yield stress (*σ*_y_) = 2 Pa, slip exponent (*s*) = 1, and slip-coefficient (*β*) = 1 × 10^4^ Pa s^*s*^/m^*s*^, flow index (*n*) = 1, and consistency index (*k*) = 8 × 10^−3^ Pa s^*n*^ for different the slip yield stress (*σ*_s_) as: (a) *σ*_s_ = 0; (c) *σ*_s_ = 1 Pa, respectively. Finite slip yield stress shown in Fig. S3(c)† is very similar to 26 °C and 27 °C data for our data shown in Fig. 3(c and d). The above arguments are also valid for investigation of the influence of slip exponent (*s*) variation on the flow curve with *σ*_s_ = 0.5 Pa, *σ*_y_ = 2 Pa, *β* = 1 × 10^4^ Pa s^*s*^/m^*s*^, *n* = 1 and *k* = 8 × 10 ^3^ Pa s^*n*^: (b) *s* = 1; (d) *s* = 2. One can observe that for *s* = 1 (Fig. S3(b)†), the flow curves show a similar trend as of Fig. 3(c and d). Therefore, inspired by the above discussion, we realized that the HB model fails to explain the data due to wall slip. Therefore, we performed more careful experiments to confirm the presence of wall slip in our system, discussed in the following section.

### Confirmation of the presence of wall slip in the system

**Fig. 4:**
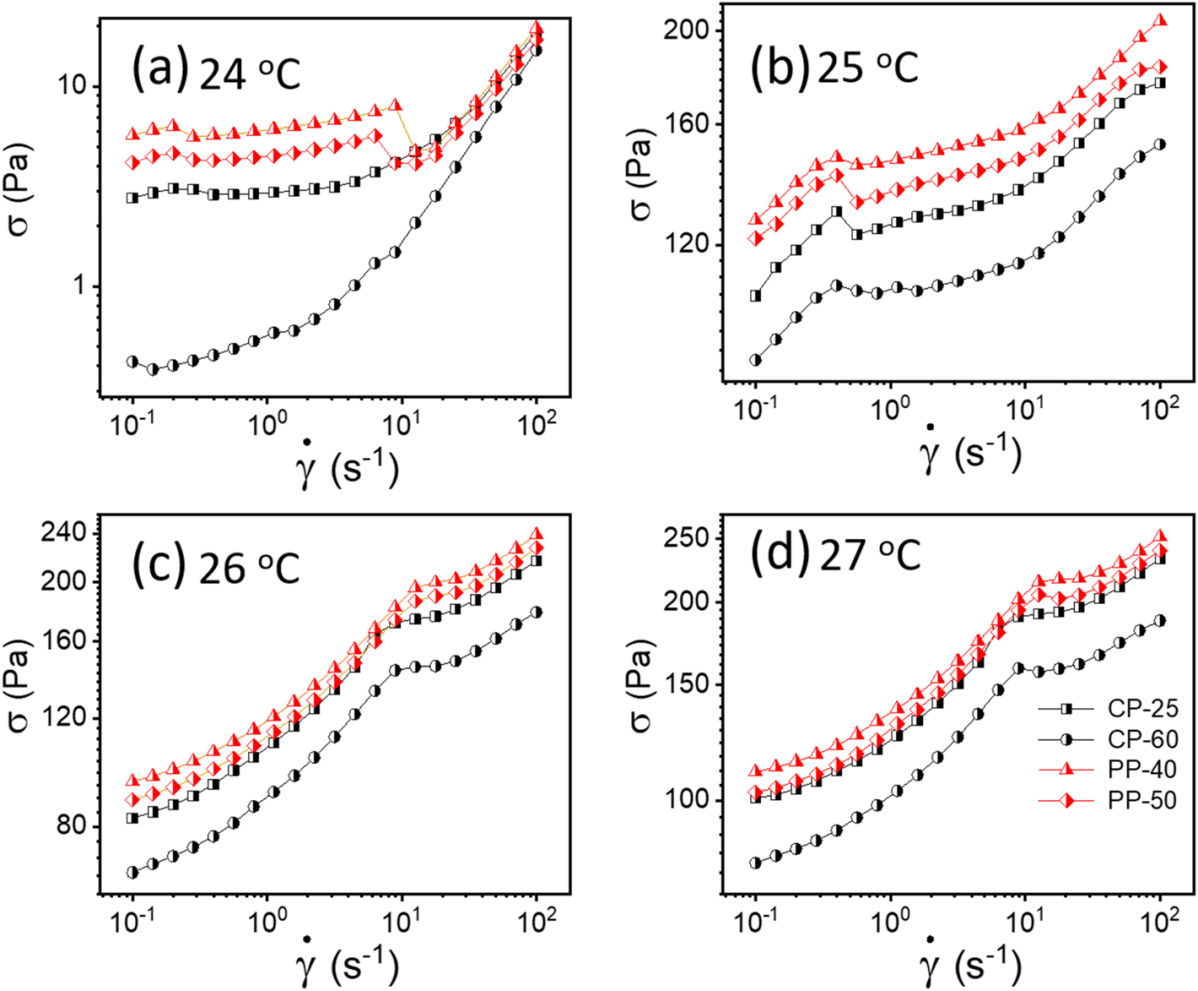
(a–d) Flow curves for 20% PF127 in different geometries (cone plate (CP) and parallel plate (PP)) at different temperatures varying from 24 to 27 °C, respectively.

To verify whether apparent wall slip exists in our system, we have conducted additional tests using various rheometer geometries, including parallel plate (PP) and cone and plate (CP). The literature claims that rheological measurements do not depend on measuring geometry; however, these values would vary if a wall slip is present.^13,50^ As seen in Fig. 4, we observed a significant change in the flow curves from our experiments with various geometries, confirming that the sample experiences wall slip during rheological measurement. One possible reason is that in cone and plate geometry, the slip tends to occur primarily at the outer edge of the cone, where the sample comes in contact with the the stationary plate.^51^ This can lead to distortion in the flow profile, affecting the measured rheological properties such as viscosity and shear stress. In parallel plate geometry, wall slip typically occurs at both surfaces. Depending on the severity of the slip, it can significantly influence the flow behavior, especially at lower shear rates where slip effects are more pronounced.^51^

As discussed, wall slip usually occurs in solids due to inadequate friction on the smooth surface. After 24 °C, a phase transition occurs from sol–gel for 20% PF127, and at higher temperatures, the sample shows solid-like behavior, as confirmed by Fig. 4. Therefore, the signature of wall slip is seen at higher temperatures. As a result, the sample deviates from the HB Model. Using a rough surface to apply more friction and ignore the slip is the proper way to deal with it. However, there was no other geometry that we could have used to prevent slip because of experimental constraints. Consequently, we have used Mooney's traditional method to calculate the true yield stress corrected from slip using different measurements at different parallel plate (PP) geometry gaps.

### Measurement of true yield stress corrected from slip by Mooney's plot using parallel plates

**Fig. 5:**
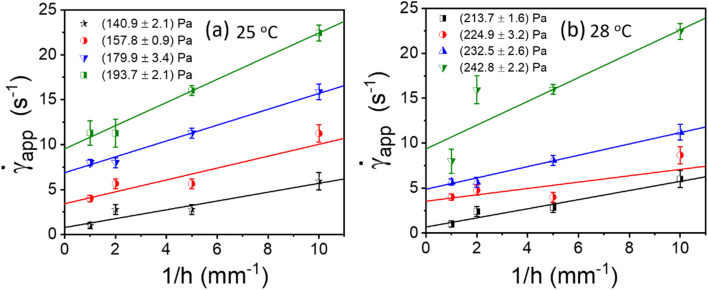
(a and b) Mooney's plots for PP-40 geometry between apparent shear (*γ̇*_app_) rate and reciprocal of the gap between two parallel plates (1/*h*) for different shear stress at different temperatures from 25 to 28 °C, respectively. Different colour lines represent the straight line fit to each data set for different shear stress values, and the symbols and error bars represented here are the mean and standard deviation of three sets of measurements in each gap size.

According to the literature, in the presence of wall slip, the shear rate measured by the rheometer differs from the true shear rate and is defined as the apparent shear rate.^23,30,31,47,52^3
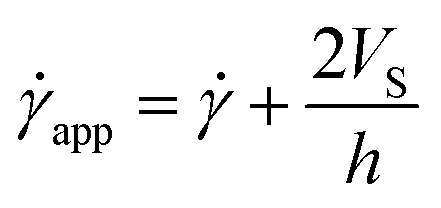
where, *γ̇*_app_ is the apparent shear rate, *γ̇* is the true shear rate corrected from slip and *V*_S_ is the apparent wall-slip velocity, and *h* is the separation gap between the parallel plates. The true yield stress (*τ*_true_) can be calculated from the plot between (1/*h*) and *γ̇*_app_ from eqn (3). A straight line gives the slope (2*V*_S_) and intercept (*γ̇*). This is typically known as Mooney's plot. As discussed in previous literature, we have first performed steady-state flow curves at different gap sizes varying from (0.1, 0.25, 0.5, and 1 mm). Then, the shear stress value and corresponding shear rate values were taken from each plot and average values (the error bar represents the mean and standard deviation of all 3 measurements in each gap size). As seen in Fig. 5 and S4,† we have plotted these curves for various temperatures and shear stresses. The fitted line has a slope of 2*V*_S_; the slip velocity and shear stress value are represented by half of the slope, and for any intercept (*γ̇*∼0), the corresponding shear stress is known as dynamic yield stress.^23,30,31,47,52^Fig. 5(a) and (b) represent Mooney's plot for 25 °C and 28 °C for different shear stress values. We observed that from Fig. 5(a) and (b) shear stress values (140.9 ± 2.1) Pa and (213.7 ± 1.6), respectively, the intercept almost approached zero; therefore, we defined these values as yield stress at that temperature. In Fig. S4,† we have shown Mooney's plot at 26–30 °C, respectively, and calculate the corresponding yield stress values.

Though this method has traditionally been used to calculate the true yield stress of a material, eliminating the slip during the measurements, this method is labor-intensive, and a large sample is required for repetitive experiments for different gaps of rheometric geometry. Therefore, to overcome these drawbacks, we followed a second approach, where we included the slip boundary conditions in the HB equation, modified the HB equation in the form of the famous Windhab model equation, and calculated the true yield stress and slip yield stress.

### Calculation of true yield stress by including slip boundary condition in HB model

**Fig. 6:**
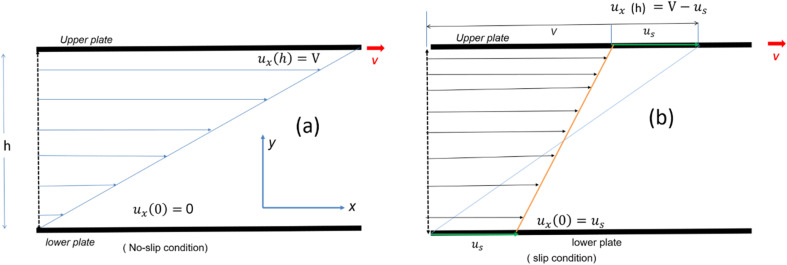
Schematic representation of velocity profile between two parallel plates with a gap (*h*) for various boundary conditions (a) no slip (b) slip boundary condition, respectively.

Consider a simple plane coquette shearing flow between two parallel plates with a gap (*h*), as shown in Fig. 6(a). The lower plate is fixed, and the upper plate moves at a constant velocity (*V*) along the *x*-direction. If the density of the fluid is (*ρ*) and the fluid velocity vector is given as ***u*** = ***u***(*u*_1_, *u*_2_, *u*_3_) where bold letter denote the vector representation and *u*_1_, *u*_2_, *u*_3_ are the *x*, *y*, and *z* components of the fluid velocity vector (***u***), then the equation of continuity (conservation of mass)^14,53^ is given as4
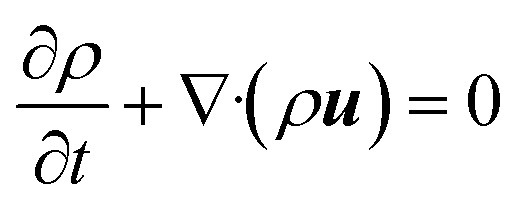


Now, for steady-state conditions and incompressible fluid (*ρ* is constant), the above equation will reduce to

5 ∇·***u*** = 0

Similarly, the equation of motion (conservation of momentum), famously known as the Navier–stokes equation,^53^ is given as6
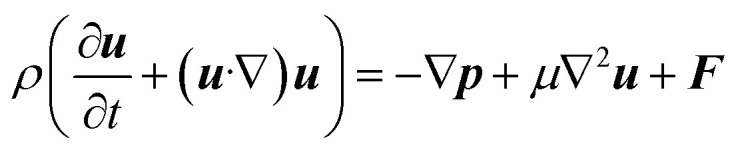
where *ρ* is the fluid density, *u* is the fluid velocity, *t* is time, *p* is the pressure, *μ* is the coefficients of viscosity, and *F* represents body forces per unit volume (*i.e.*, external forces such as gravity or electromagnetic forces).

Now, since we consider the flow is in between two plates, the velocity of the fluids in one direction, *i.e.*, along the *x*-direction, and no motion in the *y* and *z*-direction. Then, the velocity of the fluids can be represented as ***u*** = ***u***(*u*_1_, 0, 0), and since we consider a simple plane coquette shearing flow, there is no pressure gradient, *i.e.*, ∇*p* = 0, and we also assume that there exist no external forces (*F* = 0) using these conditions eqn (5) and (6) can be reduced to following,

Eqn (5) can be written as
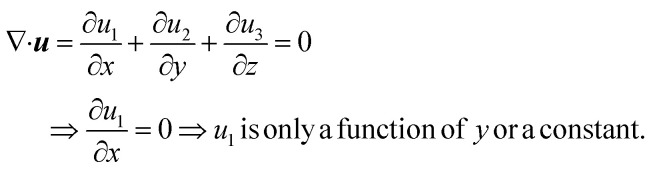


Similarly, eqn (6) reduced to only the *x*-component momentum equation, *i.e.*
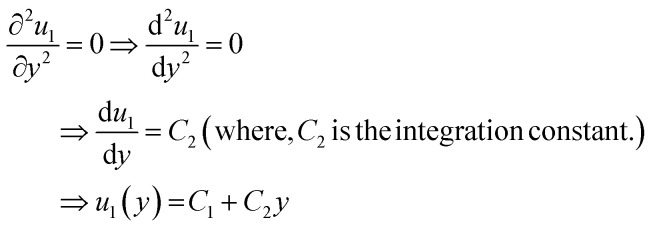


For simplicity, we drop the subscript and write *u* instead of *u*_1_

7 ⇒*u*(*y*) = *C*_1_ + *C*_2_*y*

Eqn (7) represents the velocity of the fluids between parallel plates. If we apply a no-slip boundary condition (velocity of the fluids and plate are same), *i.e.*, for a lower plate which is fixed *u*(0) = 0 and the upper plate moving with velocity (*v*) so, *u*(*h*) = *v*. Applying this to the boundary condition in eqn (7), we arrived at,8
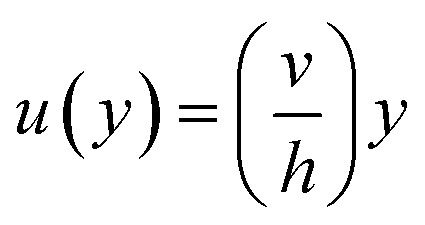


This gives a linear velocity profile for no-slip boundary conditions and is shown in Fig. 6(a), if the shear rate (*γ̇*) is linear, then this can be defined as9
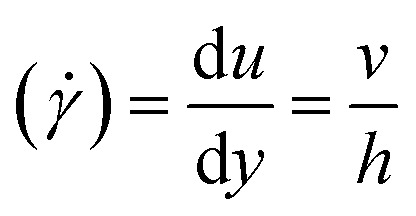


If we assume a monotonic slip law (*i.e.*, slip velocity is the same for both plates), the boundary conditions, *i.e.*, *u*(0) = *V*_S_ and *u*(0) = *v* − *V*_S_

Applying the slip boundary condition eqn (7) has the following form.10
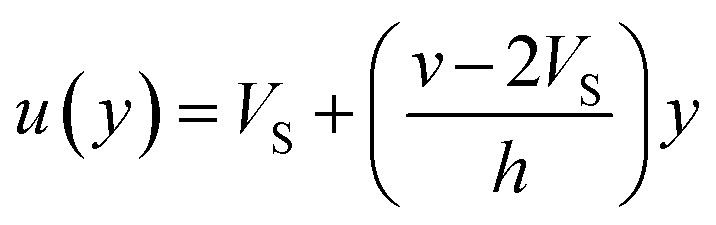


The velocity profile is still linear but slightly shifted, as shown in Fig. 6(b), and now, the true shear rate can be defined as11
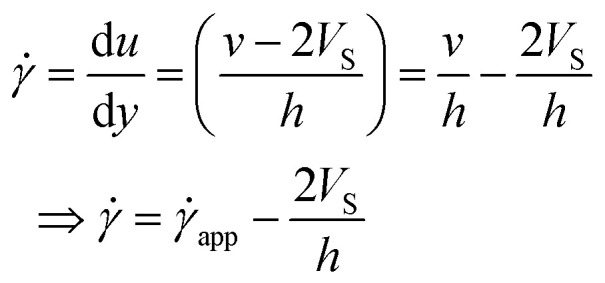


Experimentally, the shear rate is measured as the apparent shear rate,12
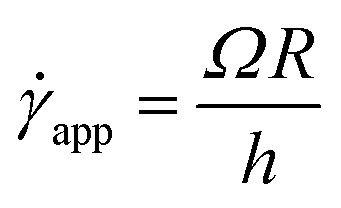
where, *Ω* is the rotational speed of the rheometric geometry, *R* is the radius of the rheometric plate, and *h* is the gap between the parallel plates.

Now, using eqn (11) in HB model*σ*(*γ̇*) = *σ*_0_ + *kγ̇*^*n*^

The modified HB model, including the slip boundary condition, has the following form:13
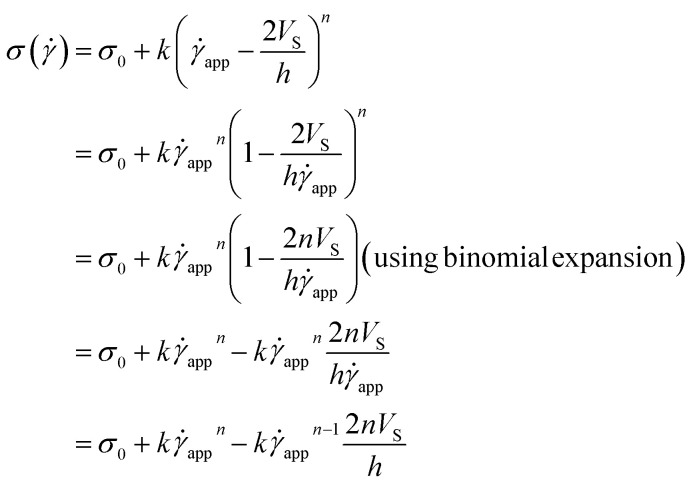
14
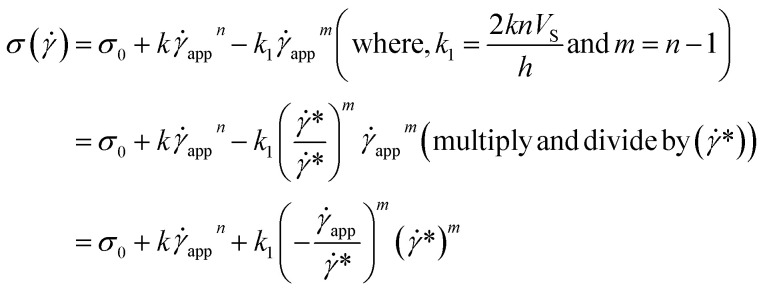
15
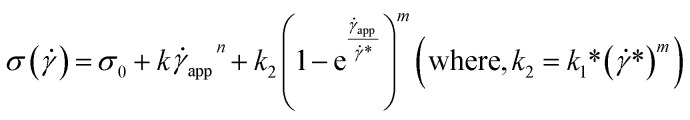


This equation is well known in the form of the Windhab model with additional power terms *m* and *n* (when *m* = *n* = 1, eqn (15) is reduced to the Windhab equation). Therefore, we defined this as a modified Windhab equation. To avoid confusion, we used *γ̇*_app_ as only *γ̇* and if we write eqn (15) with some redefined constant inspired by the Windhab model, then the equation becomes16
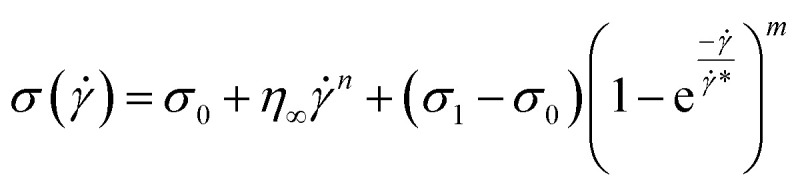
where, *k* = *η*_∞_ and *k*_2_ = (*σ*_1_ − *σ*_0_) and eqn (16) very well fits our data. But from eqn (16), we found two yield stresses (*σ*_0_, *σ*_1_) and also two exponents (*n* and *m*) and *** is a normalization constant. We have taken some arguments from different literature to understand each term's physical meaning and generalization of the approach to any system.

**Fig. 7:**
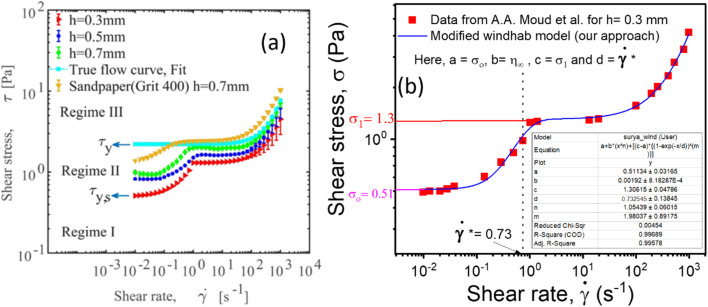
(a) Flow curves of Kaolin clay in a parallel plate geometry with different gaps (adapted and reproduced with permission from ref. 47) and (b) data taken from (a) and plotted with eqn (16). The red and pink lines are the extrapolation of yield stress values found from fitting the vertical dotted line represented by the transition shear rate obtained from the fitting eqn (16).

Consequently, to validate our approach generalized to any system, we took help from arguments discussed in Moud *et al.*,^47^ where they discussed wall slip in kaolin clays, as shown in Fig. 7(a) and discussed different regimes and their physical significance. Then, we fitted eqn (16) to the data from Moud *et al.*,^47^ as shown in Fig. 7(a), and we found that the equation fitted very well to the data. Here *σ*_0_ value lies in Regime-II, defined by Moud *et al.*^47^ in Fig. 7(a), which is nothing but the slip yield stress and *σ*_1_ lies in Regime-III, which is the true yield stress. Therefore, from fitting parameters, we found that *σ*_0_, is the slip yield stress, and *σ*_1_, is the true yield stress, and ***, is transition shear rate. When observing the flow curves, we see slope changes from low shear to higher shear rates after some shear rate. Here,*** represents the corresponding shear rate and is defined as the transition shear rate, and (*n* and *m* are slip and power-law exponents, respectively). Therefore, we write the eqn (15) as17
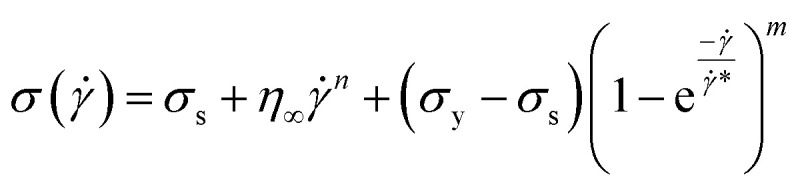
where, *σ*_s_ is slip yield stress and *σ*_y_ is the true yield stress, and all other terms are the same as defined previously. In all the flow curves we discussed, the data ranges from 0.1 to 100 s^−1^, and to verify that our approach is not limited to any range, we also fitted data for a large range, *i.e.*, from 0.01 to 1000 s^−1^, and is shown the Fig. S5.† We compared the values with the conventional Mooney's method to verify the true yield stress estimated from our method, in Table 2.

**Fig. 8:**
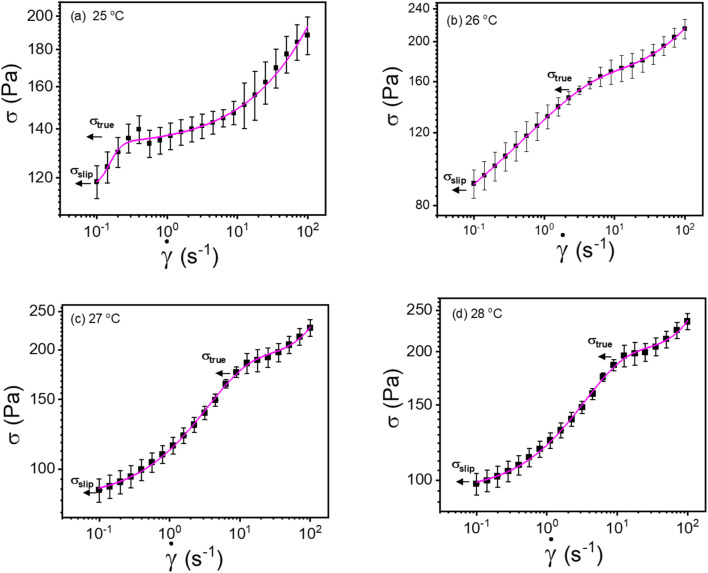
(a–d) Modified Windhab model (eqn (17)) fitting of flow curves for 20% PF127 at different temperatures from 25 to 28 °C, respectively (shear rate ranges from 0.1 to 100 s^−1^).

**Table 2:** Comparison of different types of yield stresses from our approach and Mooney's method

Temperature (°C)	Comparison of yield stress from various methods, *σ*_y_ (Pa)	
	Modified Windhab model fit (our approach) (Fig. 8)	Mooney's plot (γ̇_app_*vs.* 1/*h*) (Fig. 5)
25	138.3 ± 4.5	140.9 ± 2.1
26	160.8 ± 3.3	172.9 ± 2.9
27	201.6 ± 7.7	202.5 ± 3.8
28	213.5 ± 7.9	213.7 ± 1.6
29	223.8 ± 8.1	221.1 ± 2.9
30	230.7 ± 8.3	228.8 ± 1.5

### Temperature-dependent dynamic yield stress

**Fig. 9:**
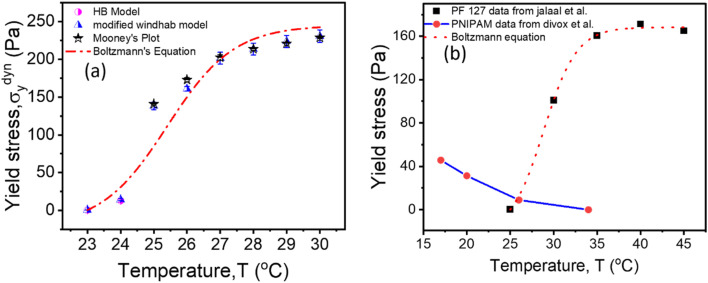
(a) Comparison of dynamic yield stress values obtained from different methods for 20% PF127. The data fits Boltzmann's equation (eqn (18)). (b) The temperature-dependent yield stress of reported data for 20% PF127 taken from Jalaal *et al.*^49^ is fitted with eqn (18), and PNIPAM gels are from ref. 43.

Fig. 9(a) shows the yield stress obtained from various methods plotted as a function of temperature. We found that yield stress increases as a function of temperature, and the experimental data following a sigmoid curve can be fitted by an empirical equation in the form of the Boltzmann equation^54^ as follows,18
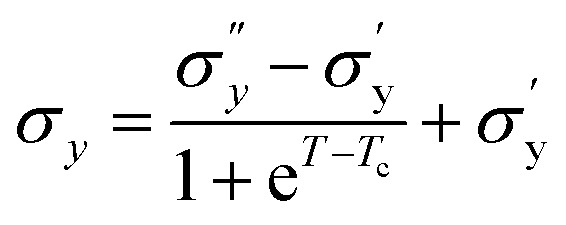
where, 
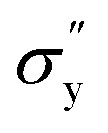
 is yield stress plateau at low temperatures and 
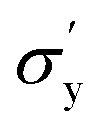
 yield stress plateau at high temperatures and *T*_c_ is the critical temperature where the yield stress increases exponentially. The yield stress values gradually increase as shown by the plateau, in this case. The increasing value of yield stress for PF127 is consistent with Jalaal *et al.*'s study^49^ at different temperatures at 5 °C intervals but doesn't formally report any trend, and as discussed earlier near the sol–gel transition temperature, each 1 °C interval is important; therefore we carefully calculated the yield stress and found the trend. The increasing trend can be understood by the mechanism suggested by Suman *et al.* that the individual micelle size may increase and can form a glassy state at higher temperatures can account for the exponential increase in the yield stress at higher temperatures.^55^ In the current study, we compared the results with other thermoresponsive polymer poly(*N*-isopropyl acrylamide) (PNIPAM) and found some interesting and contradictory results.

When comparing the yield stress for other conventional thermoresponsive polymers such as PNIPAM, whereas the temperature increased, a noticeable decrease in yield stress was shown in Fig. 9(b) (data are taken from Divoux *et al.*^43^ and plotted). This phenomenon can be attributed to the coil-to-globule transition experienced by PNIPAM, where hydrophobic interactions cause the polymer chains to collapse from extended coils to compact globules. Consequently, the overall hydrodynamic volume of PNIPAM decreases, leading to reduced intermolecular entanglements and a less dense gel network. This decrease in network density results in a lower resistance to deformation, hence the observed decrease in yield stress with increasing temperature.^56–58^ In contrast, Pluronic PF127 exhibited increased yield stress with increasing temperature. Above its critical micelle temperature *T*_CMT_ (around 15–30 °C), Pluronic PF127 undergoes micelle formation, where hydrophobic PPO blocks assemble into micellar cores surrounded by hydrophilic PEO shells. The increase in temperature leads to enhanced micelle aggregation and packing, resulting in the formation of a denser gel network. This densification of the gel network corresponds to an increase in yield stress as the gel becomes more resistant to deformation. The contrasting temperature-dependent yield stress behaviors of PNIPAM and Pluronic PF127 highlight their distinct gelation mechanisms. While PNIPAM's coil-to-globule transition decreases yield stress, Pluronic PF127's micelle formation leads to an increase in yield stress with increasing temperature.^58^ The biomedical field has used body temperature as the transition temperature for two to three decades. Examining the yield stress concerning temperature can provide an additional understanding of the rheological characteristics of PF127 in diverse drug delivery applications and transport phenomena.

## Conclusion

This study thoroughly investigated the yield stress behavior of PF127 at various temperatures using the Herschel–Bulkley model and the modified Windhab model, shedding light on the rheological characteristics of the thermoresponsive polymer. The HB model effectively described the data at lower temperatures (23 °C to 25 °C) but failed at higher temperatures (26 °C and 27 °C) due to wall slip. This failure was indicated by a slope change in the mid-shear range with a kink around a shear rate of 10 s^−1^, and unrealistic yield stress values at these temperatures.

Further tests proved the presence of wall slip using flow curves depending on rheometric geometry, which led to the adoption of the modified Windhab model to address the limitations of the HB model. The flow curves at higher temperatures were more accurately fitted (with higher *R*-square values) by this wall slip-accounting model. The temperature-dependent yield stress increased significantly as the results showed, following a sigmoid curve that could be explained by an empirical equation like the Boltzmann equation. Comparative analysis with other thermoresponsive polymers, such as PNIPAM, highlighted the distinct gelation mechanisms and temperature-dependent yield stress behaviors of PF127. Unlike PNIPAM, which shows a decrease in yield stress with increasing temperature due to the coil-to-globule transition, PF127 exhibited an increase in yield stress attributed to enhanced micelle aggregation and network densification at higher temperatures.

This research emphasizes the importance of considering wall slip effects in rheological measurements and provides a robust framework for understanding the temperature-dependent rheological properties of PF127. The findings have significant implications for using PF127 in biomedical applications, particularly in drug delivery systems where temperature-sensitive gelation is crucial. Future research should explore applying these models to other complex fluids and further investigate the mechanisms underlying the observed temperature-dependent behaviors.

## Data availability

The data supporting this study's findings are available from the corresponding author upon reasonable request.

## Conflicts of interest

There are no conflicts to disclose.

## Supplementary Material

RA-014-D4RA04825C-s001
