# Supplementary material for: Temperature-dependent yield stress and wall slip behaviour of thermoresponsive Pluronic F127 hydrogels
Source: RSC Adv. 2024 Jul 29;14(33):23772–84. doi: 10.1039/d4ra04825c (PMC11284911; doi:10.1039/d4ra04825c)
Supplement: RA-014-D4RA04825C-s001 [file RA-014-D4RA04825C-s001.pdf]

Electronic Supplementary Material (ESI) for RSC Advances.
This journal is © The Royal Society of Chemistry 2024

Supplementary Information

Temperature-dependent yield stress and wall slip behaviour of thermoresponsive Pluronic F127 hydrogels

Surya Narayana Sangitra and Ravi Kumar Pujala*

Soft and Active Matter group, Department of Physics, Indian Institute of Science Education and Research (IISER) Tirupati Yerpedu, Tirupati - 517619, Andhra Pradesh, India.

Corresponding author's E-mail address: pujalaravikumar@iisertirupati.ac.in

S1. Flow curves of PF127 solutions at different temperatures

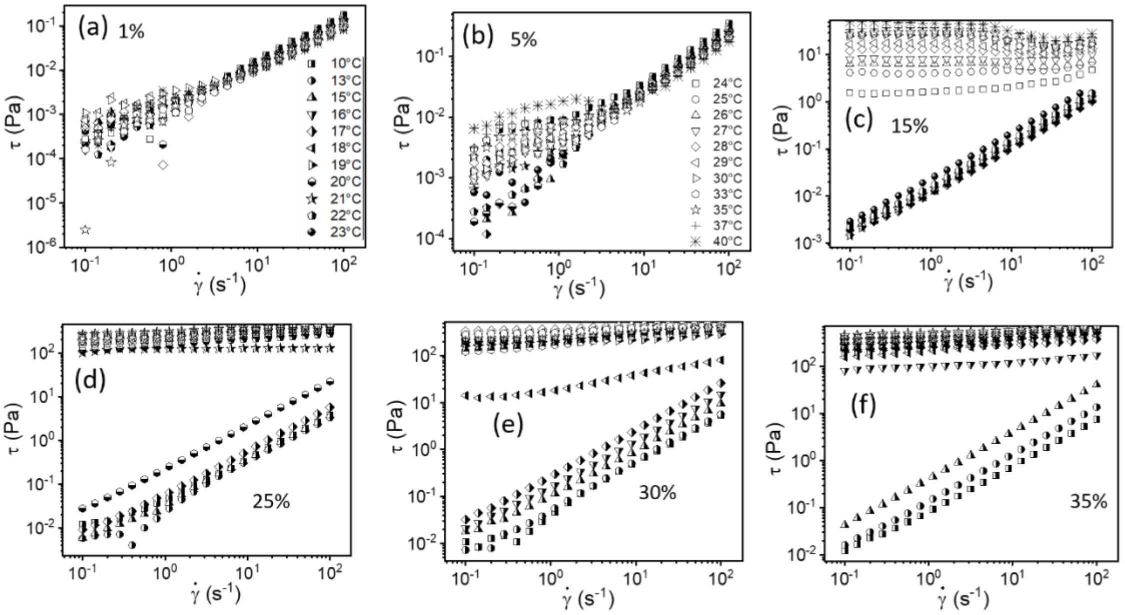

Figure S1. (a)-(f) Flow curves of PF127 at different temperatures varying from 20 °C to 30 °C with 1 °C intervals for 1%, 5%, 15%, 25%, 30% and 35%, respectively.

The flow curves, i.e. Shear stress (τ) Vs. Shear rate ($\dot{\gamma}$), for different concentrations at different temperatures is shown in Figure S1. One can observe the yield stress behavior after 15% PF127; the transition temperature decreases as a function of concentrations. As per observation, for 15%, the T_{SG} is 24 °C, and for 20%, 25%, 30%, and 35% are, 23 °C, 21 °C, 18 °C and 16 °C, respectively.

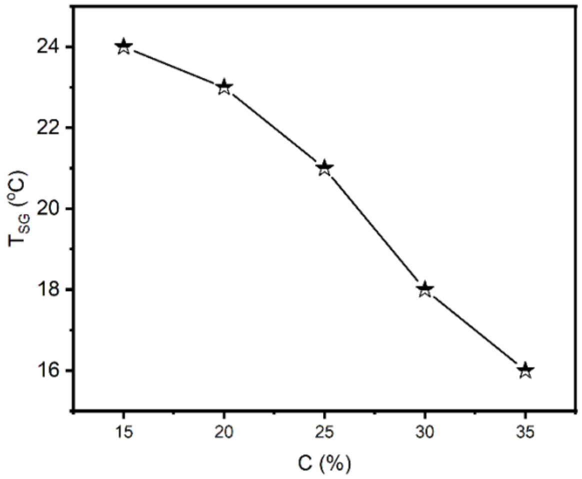

Figure S2: Sol-gel transition temperature (T_{SG}) is plotted as a function of different concentrations(C %(w/v)) of PF127.

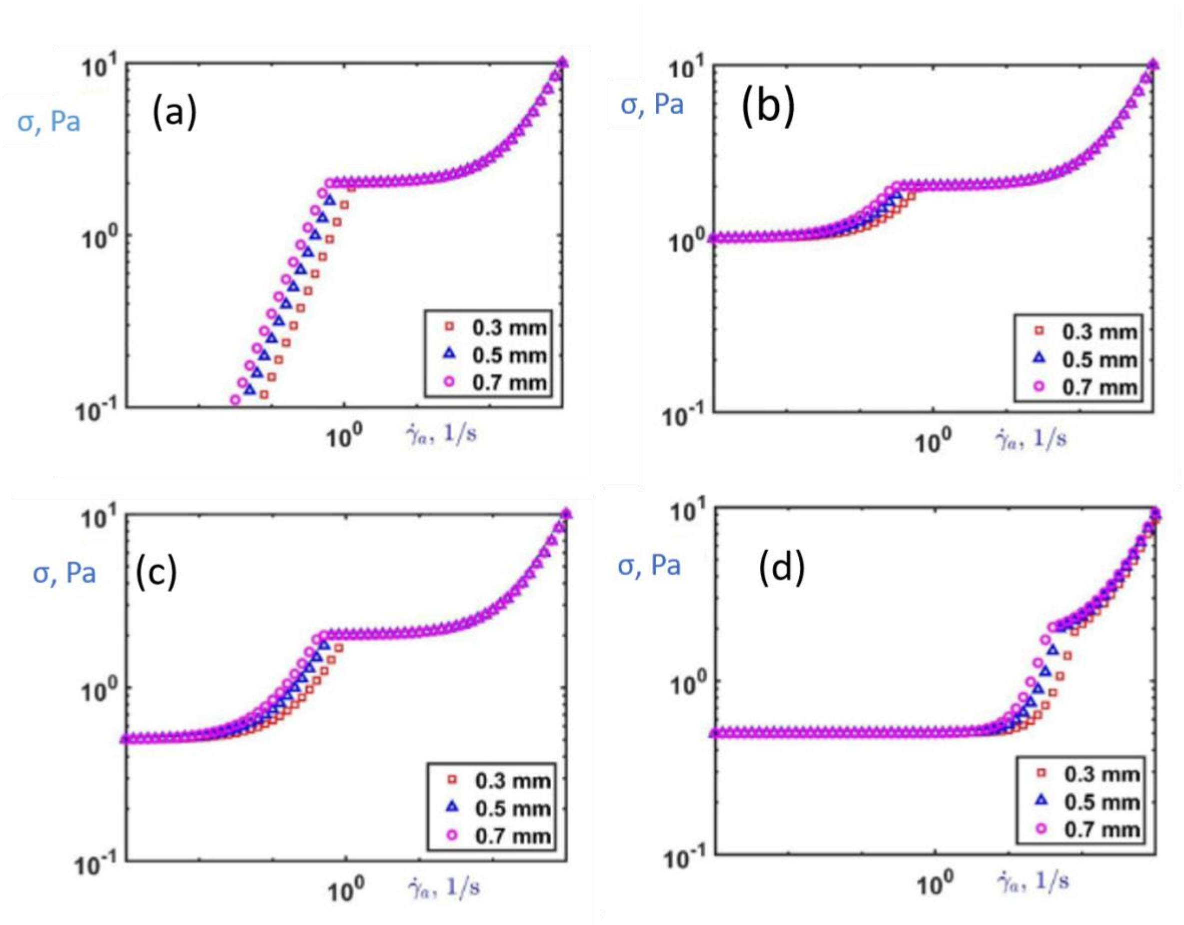

Figure S3: shows **(a)** and **(b)** represents flow curves (shear stress ($\boldsymbol{\sigma}$) vs. shear rate ($\dot{\boldsymbol{\gamma}}$)) generated numerically for HB fluids in case of with the yield stress ($\boldsymbol{\sigma_y}$) = 2Pa, slip exponent (s) = 1, and slip-coefficient (β) = $1 \cdot 10^4$ $Pa \cdot^{s}$ /m^s, flow index *(n)* =1, and consistency index *(k)*=

$8\cdot10^{-3}$Pa·s^n for different the slip yield stress $\boldsymbol{\sigma_s}$ = 0 and $\boldsymbol{\sigma_s}$ = 1Pa respectively and **(c)** and **(d)** shows variation on the flow curve with $\boldsymbol{\sigma_s}$ = 0.5Pa, $\boldsymbol{\sigma_y}$ = 2Pa, β = $1\cdot10^4$ Pa·s^s /m^s, n = 1 and k = $8\cdot10^{3}$Pa·s^n: with different slip exponents s = 1 and s = 2, respectively. The Figures are adapted and reproduced with permission from Ref.[47].

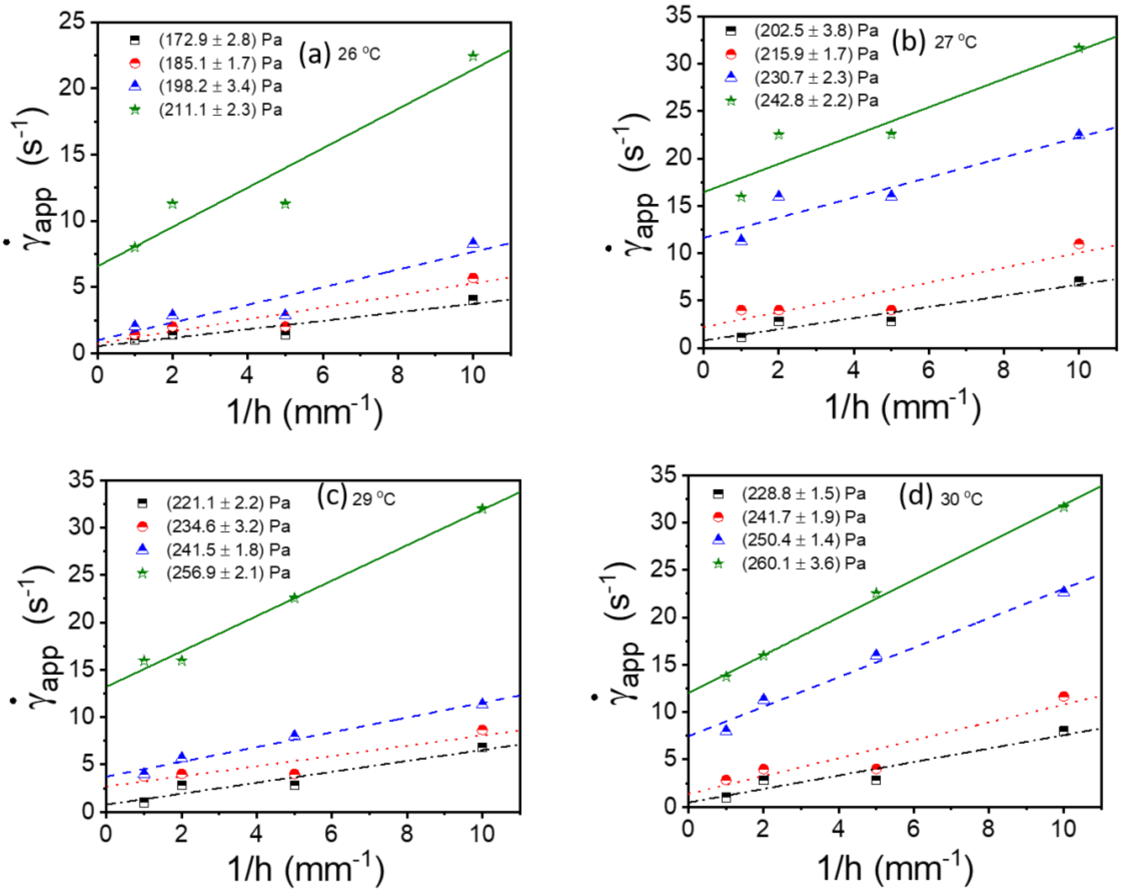

Figure S4: (a)-(d) represents Mooney's plots for PP-40 geometries between apparent shear ($\dot{\gamma}_{app}$) rate and reciprocal of the gap between two parallel plates (*1/h*) for different shear stress at different temperatures from 26 °C – 30 °C, respectively.

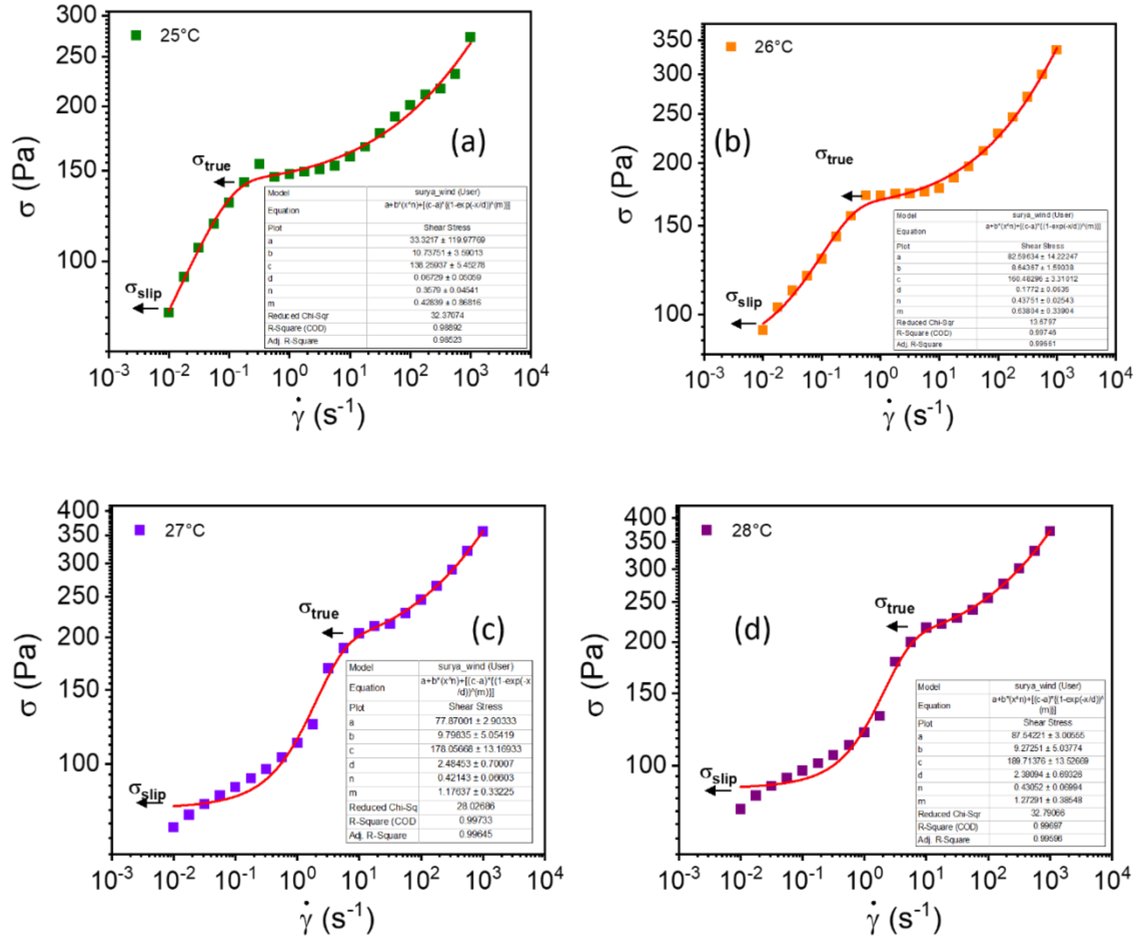

Figure S5: (a)-(d) represents the Windhab model (equation 15) fitting with flow curve data for 20% PF127 at temperatures at temperatures varying from 25 ^{o}C – 28 ^{o}C, respectively (for data set, 0.01 to 1000 s^{-1}).
